# The role of quantitative EEG biomarkers in Alzheimer’s disease and mild cognitive impairment: applications and insights

**DOI:** 10.3389/fnagi.2025.1522552

**Published:** 2025-04-23

**Authors:** Yue Yuan, Yang Zhao

**Affiliations:** Department of Neurology, The First Hospital of Jilin University, Changchun, Jilin, China

**Keywords:** AD, Alzheimer’s disease, MCI, mild cognitive impairment, QEEG, quantitative electroencephalography, diagnosis, prediction

## Abstract

Alzheimer’s disease (AD) is characterized by the pathological accumulation of amyloid plaques and hyperphosphorylated tau proteins, leading to disruptions in synaptic transmission and neural circuit alterations. Despite advancements in therapies to delay disease progression, there is a pressing need for simple, non-invasive, and accessible biomarkers to evaluate their effectiveness. Quantitative electroencephalography (qEEG), a computational method for quantifying brain electrical activity, is increasingly applied in AD research. We highlight the application of qEEG biomarkers, including power spectrum analysis (oscillatory activity within frequency bands), functional connectivity (coherent neural couplings) and effective connectivity (directional neural interactions), microstates (brief, stable states of the brain network), and non-linear analyses (e.g., entropy and EEG network analysis). These biomarkers can reflect real-time neural dynamics, making them ideal tools for diagnosis and monitoring the progression AD and mild cognitive impairment (MCI). It has been shown that decreased α power and increased θ power within the qEEG spectrum correlate with enhanced AD severity. Data from microstate analysis have demonstrated significant variations in temporal dynamics in patients with AD. Non-linear measures, such as entropy, have identified marked reductions in neural complexity in AD and MCI patients, indicating that they may serve as early diagnostic markers. Compared to traditional neuroimaging techniques, such as magnetic resonance imaging (MRI) or positron emission tomography (PET), qEEG is known to be cost-effective and facilitates real-time monitoring. Overall, qEEG biomarkers are promising for advancing AD research due to their non-invasive nature, affordability, and ability to capture real-time neural activity. Integrating qEEG with multimodal neuroimaging and clinical profiles may facilitate earlier identification and precision therapies. Future research should focus on standardizing protocols, validating biomarkers across diverse cohorts, and exploring their potential in large-scale clinical trials.

## 1 Introduction

Alzheimer’s disease (AD) is a progressive neurodegenerative disorder characterized by memory impairment, cognitive function decline, decline in daily living ability, and mental behavior abnormalities (Knopman et al., 2019). As the most common form of dementia, AD accounts for 60–80% of all dementia cases worldwide (Lynch, 2020). According to the Alzheimer’s Association International, every 3 s, a new case of AD is diagnosed worldwide. Over 130 million AD patients exist worldwide as of 2050 (Alzheimer’s Disease International, 2013). Clinically, mild cognitive impairment (MCI) presents a transitional state between normal aging and dementia. It is classified into two types: the amnestic MCI (aMCI), characterized by memory impairment, and the non-amnestic MCI, affecting other cognitive domains. Generally, the aMCI type is the most frequent, and 90% of patients may progress to AD (Anderson, 2019). AD patients are more likely to require extended care, imposing significant economic and social burdens on patients. Therefore, early diagnosis and implementation of appropriate interventions are recommended to prevent disease progression and alleviate the burden and stress on caregivers. The currently used early diagnostic procedures for AD include techniques such as Magnetic resonance imaging (MRI), positron emission tomography (PET-CT), cerebrospinal fluid (CSF) biomarker analysis, and neuropsychological evaluation tools. Notably, CSF analysis is an invasive procedure, while PET-CT is expensive and inconvenient in certain clinical situations. Therefore, it is crucial to identify low-cost, non-invasive, sensitive, specific, and convenient biomarkers for AD diagnosis. Electroencephalography (EEG) is a promising digital biomarker model that is not only non-invasive, mobile, and convenient compared to other detection techniques, but also exhibits low spatial resolution (centimeters) and high temporal resolution (milliseconds), and its abnormal changes are often earlier than structural imaging changes.

Recently, many researchers have suggested regulatory requirements and guidelines for including EEG biomarkers in AD research and management (Babiloni et al., 2021). Unlike event-related potentials (ERPs), qEEG does not require stimulus-related devices or behavioral recordings, making it simpler to implement in clinical settings. It is especially suitable for large-scale screening and disease progression monitoring, and it has been used to differentiate AD from other forms of dementia, such as frontotemporal dementia or dementia with Lewy bodies (DLB) (Caso et al., 2012; Engedal et al., 2015; Iannaccone et al., 2023). Therefore, qEEG is a valuable marker for AD. In this paper, we summarize the application and progress of qEEG in AD research, highlighting its potential as a diagnostic and monitoring tool and identifying gaps for future exploration.

## 2 Common analytical metrics of qEEG in AD

QEEG recordings help clinicians to identify changes in neuronal synaptic activity, topographic distribution, and synchrony in AD patients (Smailovic and Jelic, 2019; Colom-Cadena et al., 2020). Generally, neuronal synaptic activity is analyzed through spectral analysis of EEG recordings to determine the frequency and amplitude of brain wave patterns. On the other hand, topographic distribution is explored using spatial mapping techniques, such as topographical plots, which display the electrical activity in various scalp regions. Synchrony, as measured using coherence and phase-locking value (PLV) analysis, can indicate the correlation in oscillatory activities among various brain areas.

Besides these conventional measurements, novel developments underscore the significance of non-linear analysis, including entropy measures (e.g., sample entropy, multiscale entropy) and higher-order spectral analysis (e.g., bispectral analysis), which capture the complexity, irregularity, and phase coupling of EEG signals. Effective connectivity analysis applies methods like Granger causality or dynamic causal modeling and is more sophisticated than functional connectivity by modeling causal influences between brain regions. Additionally, EEG connectivity networks and graph-theory-based approaches analyze the brain as a complex network by measuring network characteristics such as node centrality and clustering coefficient. New approaches such as Graph New approaches, such as graph neural networks (GNNs), can also be employed by reconstructing spatiotemporal dynamics, providing deeper insights into the disrupted network topology and organization in AD. These approaches provide important insights into the general effects of the disease on the brain’s functionality and connectivity.

### 2.1 Frequency domain analysis

#### 2.1.1 Power spectrum analysis

Power spectrum analysis applies computational techniques such as the fast Fourier transform (FFT) to convert time-domain EEG amplitude fluctuations into frequency-domain power distributions. This approach is performed using the following protocol: Firstly, EEG signals are recorded using electroencephalography machines, which are then converted from the time domain to the frequency domain through FFT. This allows the measurement of brain waves of varying frequencies. The signals are then divided into multiple frequency bands (e.g., δ, θ, α, β, and γ waves), each reflecting the intensity of brain electrical activity under a specific state.

Absolute power quantifies the magnitude of power in a specific frequency band (e.g., α or β waves) within a specified duration. It is the actual “energy” or amplitude of said frequency band in the EEG. Higher absolute power indicates more vigorous brain activity in that frequency range. Relative power is the proportion of power in a particular frequency band relative to the total power in a given brain region. Unlike absolute power, relative power offers a more stable measure by accounting for global variations in overall brain activity. Studies on cognitive impairment often use relative power ratios, such as α/θ, θ/γ, α3/α2, and (δ+θ)/(α+β) relative power ratio (DTABR) (Moretti et al., 2009; Schmidt et al., 2013; Moretti, 2018; Niu et al., 2023).

Researchers have shown that AD patients present with a generalized slowing of EEG activity, indicated by high power of slow-frequency bands (δ and θ) and low power of high-frequency bands (α and β) (Farina et al., 2020). Specific relative power ratios reflecting changes in slow and fast waves, such as the α/θ ratio (Schmidt et al., 2013) and the DTABR (Niu et al., 2023), have been shown to exhibit increasing values that are helpful in the differentiation of AD and are considered significant markers for the detection of early AD risk. In aMCI patients, the most prominent EEG changes are decreased β power and increased δ and θ power (Zawiślak-Fornagiel et al., 2024), while α power tends to be less affected. The changes are strongest in the temporal region (Meghdadi et al., 2021). In relative power ratios (Moretti et al., 2009), increases in the θ/γ and α3/α2 ratios have been demonstrated to contribute to the detection of aMCI (Moretti, 2018). The underlying causes of these changes remain unclear. These patterns of spectral disorganization likely reflect a breakdown in thalamocortical oscillatory coordination and synaptic efficiency, potentially driven by Aβ/tau-mediated neurodegeneration, neuronal death, defective synapses, or compensatory processes. However, further multimodal studies integrating PET amyloid imaging and high-density EEG are needed to disentangle these cascading pathophysiological processes.

#### 2.1.2 Global field power analysis

While power spectrum analysis provides valuable insights into the distribution of brain activity across specific frequency bands, it primarily focuses on localized or regional patterns. To complement this, Global Field Power (GFP), derived from the spatial standard deviation of voltage values across all electrodes at a given time, measures global brain activity’s instantaneous spatial synchronization strength. As a key qEEG metric, GFP provides insights into brain activity’s overall strength and synchronization across frequency bands, offering a broader perspective on the global integration of neural dynamics.

Compared to normal controls (NC), AD patients exhibit increased GFP in the θ and δ bands and decreased GFP in the α band. AD patients show reduced GFP in the α and β bands compared to aMCI patients. However, no significant GFP differences were observed between MCI patients and normal controls (Huang et al., 2000), suggesting that GFP may not effectively distinguish MCI from regular brain activity. Further analysis dividing aMCI into single-domain aMCI and multiple-domain aMCI revealed no significant group differences in GFP (Smailovic et al., 2022), indicating that GFP may not be suitable as a marker for differentiating these MCI subtypes. Nevertheless, longitudinal follow-up of MCI patients demonstrated that those who progressed to AD exhibited reduced α GFP and more anteriorly localized θ, α, and β frequency sources compared to those who remained stable (Huang et al., 2000). This suggests that the anterior-posterior localization of α GFP may serve as a potential predictor for MCI progression to AD.

These dynamic shifts in GFP topography—particularly the anterior migration of α sources in progressive MCI—may reflect compensatory recruitment of neural circuits network disintegration. This suggests that GFP spatial evolution could serve as a tractable biomarker for tracking network-level adaptations during preclinical AD progression. Smaller sample sizes and methodological differences limit the generalizability of results, highlighting the necessity for standardized protocols and larger cohorts. Meanwhile, its utility in MCI is constrained by the lack of significant differences between MCI subtypes and normal controls, emphasizing the need for multimodal approaches. Most studies suffer from inadequate sample sizes and insufficient evidence to endorse GFP’s application in early MCI diagnosis. Future research should prioritize large-scale validation and investigate the integration of GFP with other biomarkers to enhance diagnostic accuracy and clinical applicability.

### 2.2 Functional connectivity analysis

GFP can reflect global changes in neural activity in patients with AD and MCI, but information regarding interregional communication is currently lacking. Functional connectivity analysis complements by assessing temporal correlations and network interactions, offering further insights into the network-level disruptions underlying cognitive impairment.

A common approach is phase synchronization, which includes coherence and phase-locking value (PLV) to measure how brain regions coordinate their activity (Bastos and Schoffelen, 2015). Studies show that AD patients have reduced coherence, especially in the α frequency band (Jelic et al., 1996, 1997; Meghdadi et al., 2021), with decreased coherence in the right central parietal lobe potentially serving as an early biomarker for aMCI (Zawiślak-Fornagiel et al., 2024). However, coherence measurements are only calculated between channel pairs and are influenced by volume conduction effects.

Synchronization likelihood (SL) is a quantitative measure of brain connectivity that assesses the dynamic interdependence between pairs or sets of EEG channels (Stam and van Dijk, 2002). In patients with AD, SL is reduced across all frequency bands and has been associated with cognitive decline (Stam et al., 2003; Stam et al., 2004; Babiloni et al., 2006). Phase lag index (PLI), which quantifies the asymmetry of the distribution of phase differences between signals to reduce the impact of volume conduction (Stam et al., 2007), reveals reduced high-frequency connectivity with increasing severity of AD but enhanced θ band connectivity compared to healthy controls (Engels et al., 2015; Briels et al., 2020). However, PLI only indexes synchronization between signals in pairs but not on the instantaneous multi-brain regions’ interactions (Smailovic and Jelic, 2019). To resolve this challenge, global field synchronization (GFS), which estimates functional synchronization for multi-channel EEG data, such that decreased α and β band synchronization and increased δ band activity characterize cognitive impairment (Koenig et al., 2005). Abnormally low GFS in frequency bands (δ, θ) may indicate AD-related synaptic dysfunction in its earlier stages (Smailovic et al., 2022).

The graph theory analysis can reveal insights into the pathology of the brain network in AD and MCI. By comparing topological properties, like mean node degree, clustering coefficient, characteristic path length, global efficiency, and local efficiency, this approach allows systematic measurement of abnormal brain network connectivity. Higher θ band node degree in the occipital lobe and lower α band clustering coefficient and local efficiency in AD patients reflect impaired information transmission and processing (Wu et al., 2024). In contrast, MCI patients show a higher average clustering coefficient than NC and lower global efficiency, indicating more local connections but less efficient global transmission (Youssef et al., 2021). These changes provide a neural network-level explanation of cognitive impairment in AD and MCI.

### 2.3 Spatial source localization analysis

Although qEEG can reveal important insights into aspects such as the spectral and connectivity of EEG signals, it has several limitations. Specifically, it cannot identify the brain location where the neural activity is generated. Low-resolution electromagnetic Tomography (LORETA) is a functional neuroimaging method that locates the sources of electrical activity in the brain through a three-dimensional model. As a standardized method (sLORETA), it precisely localizes altered neural activity and connectivity, providing deeper insights into the mechanisms of cognitive impairment in AD and MCI (Pascual-Marqui et al., 1994).

In AD patients, the parieto-occipital α source activity was decreased, while δ and θ band source activity was increased compared with healthy controls. Cortical sources of δ, θ, and α activity were significantly correlated with neuropsychological test scores (Babiloni et al., 2007; Kim et al., 2012; Babiloni et al., 2013b). In patients with amnestic MCI, the cortical source of EEG rhythm has been reported to be abnormal.

A previous study collected resting EEG data at baseline and after about 1 year in MCI subjects and estimated EEG cortical sources. They found that the posterior α1 source power on the baseline EEG of MCI subjects was between NC and AD. Analysis of EEG recordings from MCI patients at baseline and one-year follow-up showed reduced α power in the parietal, occipital, and temporal lobes at both low and high frequencies. This suggests that resting EEG α sources could be a valuable marker for tracking the cognitive decline in amnestic MCI over a year (Babiloni et al., 2014). Therefore, researchers should test the application of LORETA to estimate EEG cortical sources, thereby tract disease progression or even evaluate the effectiveness of specific drugs designed to improve cognitive impairment.

### 2.4 Microstates analysis

EEG microstate analysis detects the periodic activity of neural networks by using stable electric field topographic maps. These maps represent the two-dimensional distribution of EEG potential values recorded at all electrode points at a specific time. Spontaneous resting-state EEG recordings appear as a sequence of alternating brief brain states known as functional microstates (Smailovic and Jelic, 2019). The commonly analyzed microstate characteristics include the average duration of each microstate, incidence, coverage, and conversion rate between microstate types. These microstates remain stable for prolonged periods (typically 60–120 ms) and exhibit organizational alterations over time (Michel and Koenig, 2018), where duration is defined as the average time (in milliseconds) that a given microstate remains stable each time it occurs (Bejia et al., 2023).

Prior studies show that AD patients exhibit reduced microstate duration, increased optimal window size, and anterior shifts in the center of gravity, suggesting cognitive decline and potential diagnostic utility (Dierks et al., 1997; Strik et al., 1997). However, Schumacher et al. (2019) recently found no significant changes in microstate duration in AD, though topography alterations were observed. This discrepancy likely arises from pathological heterogeneity, where distinct Aβ/tau burden patterns differentially affect network stability, as well as methodological differences: Schumacher’s 5-class spatial template model prioritizes stable brain activity patterns, while Strik’s duration-based classification (single/multiple/longest microstates) focuses on temporal persistence (Strik et al., 1997). The choice of template number (e.g., 5-class vs. traditional 4-class methods) can affect timing metrics by changing state transitions. These methodological variations underscore the importance of standardized approaches in microstate analysis to achieve consistent and reliable results.

### 2.5 Effective connectivity analysis

Effective connectivity describes the directed influence of one neural region or network on another, whether direct or indirect. It is commonly analyzed using causality-based methods like Granger causality (GC) and directed transfer function (DTF). These methods measure the direction and strength of information flow within brain networks, offering insights into how different regions interact during cognitive processes or disease development.

Cai et al. (2017) investigated effective connectivity patterns in the executive control network (ECN) of 85 MCI patients, categorized by 24-month outcomes (reversion to normal, stable MCI, or progression to AD) and 39 healthy controls. Through independent component analysis to identify ECN nodes (e.g., dorsolateral prefrontal cortex and medial prefrontal cortex) and Granger causality analysis, distinct effective connectivity patterns were identified among MCI subgroups (Cai et al., 2017). These findings suggest that ECN dynamic impairments can differentiate MCI subtypes and serve as neuroimaging markers for early AD prediction. In another study, researchers used DTF and GC analyses to compare brain networks during visual working memory tasks in 21 MCI patients and 20 NC. MCI patients showed reduced θ-band frontal-temporal connectivity but increased frontal-occipital and parietal-occipital θ/α connectivity, suggesting compensatory mechanisms. Key regions (frontal [Fz] and parietal [Pz]) had reduced θ-band information outflow, with α-band differences mainly in the parietal lobe. These results highlight disrupted prefrontal-temporal interactions in MCI and potential compensatory adaptations (Jiang et al., 2024).

However, EEG-based effective connectivity analysis faces inherent limitations due to its poor spatial resolution, limiting its ability to localize neural generators and quantify directional information flow precisely. EEG’s susceptibility to noise contamination compounds this challenge. In contrast, fMRI and MEG are prioritized in connectivity research owing to their higher spatial resolution. Recent advances in multimodal integration, particularly EEG-fMRI/MEG integration, are overcoming these constraints by combining the temporal resolution of EEG with the spatial fidelity of complementary modalities.

### 2.6 Other non-linear analysis techniques

While linear qEEG analyses, such as power spectral analysis, GFP, and coherence analysis, capture certain aspects of brain electrical activity, they are limited in their ability to characterize the complex, non-linear dynamics of brain function fully. To address this, recent research has increasingly focused on non-linear EEG features, which offer deeper insights into the intricate patterns of neural activity. In the context of AD diagnostics, non-linear methods have proven particularly valuable, revealing that AD is associated with reduced complexity and increased regularity in spontaneous brain activity—findings that linear approaches often fail to detect (Gómez et al., 2009a,b). These developments emphasize the significance of non-linear analysis in identifying hidden biomarkers and enhancing our understanding of brain disorders.

#### 2.6.1 Entropy

Entropy is a non-linear dynamic parameter that quantifies the emergence of new information in a time series and measures the complexity of system behavior. In AD research, sample entropy (SampEn) and multiscale entropy (MSE) are the most commonly used measures.

SampEn assesses signal complexity by evaluating sequence similarity, where lower values indicate higher regularity and higher values suggest more significant disorder. EEG studies have shown a progressive decline in SampEn values with cognitive impairment (AD < MCI < HC). In contrast, relative power spectral analysis often fails to detect significant abnormalities in MCI (Tao et al., 2024), underscoring SampEn’s unique ability to capture non-linear dynamics that conventional spectral metrics miss. A recent study used SampEn, permutation entropy (PE), and Lempel-Ziv complexity (LZC) to evaluate brain entropy responses to music therapy in AD patients with different levels of dementia severity. The results showed that mild-to-moderate AD patients had higher post-stimulation entropy compared to severe AD patients (Wu et al., 2022). These findings offer valuable insights into disease progression and inform personalized music-based interventions.

MSE builds on SampEn by incorporating time-scale variability, uncovering changes in complexity across different time scales. The short-scale MSE is sensitive to high-frequency signals (e.g., α/β), while the long-scale MSE reflects low-frequency activity (e.g., θ/δ). In AD patients, short-scale entropy decreases in frontal, temporal, central, and occipital regions, while long-scale entropy increases, reflecting spectral slowing (Zúñiga et al., 2024). Furthermore, MSE and refined multiscale spectral entropy (rMSSE)—a combination of spectral and multiscale analysis—were applied to EEG data from NC, MCI, and mild/moderate/severe AD groups. Both metrics demonstrated significant differences in complexity across disease stages, especially between the transitions from NC to MCI, MCI to mild AD, and from moderate to severe AD (Maturana-Candelas et al., 2019). These findings underscore their potential for tracking neurodegenerative changes and developing biomarkers for disease progression.

#### 2.6.2 High-order analysis

Although studies using Lempel-Ziv complexity, multiscale entropy, and sample entropy have revealed reduced complexity and irregularity in AD brains, these metrics fail to capture the intrinsic high-order interactions between non-linear frequency components in EEG signals. Bicoherence analysis—a statistical method extending spectral analysis—quantifies non-linear phase coupling across frequencies. In AD patients, bicoherence analysis has revealed reduced bispectral peaks, diminished amplitudes, and decreased phase-coupling complexity (reflected by lower bispectral entropy), indicating pathological shifts toward simplified and regularized dynamics (Wang et al., 2015).

Cross-Bispectrum (CBS) can reveal the inherent complexity of a single signal and capture the interdependence and non-linear interaction between two signals. By combining traditional linear functional connections (based on cross-spectral analysis, only analyzing signals within a single frequency band) and non-linear cross-band functional connections (based on CBS analysis, capturing interactions between different frequency bands), a brain functional network is constructed, and two types of features are fused for AD classification. Results demonstrated that while single-band and cross-frequency networks achieved high diagnostic accuracy, CBS-based non-linear cross-frequency connectivity significantly outperformed linear methods (Klepl et al., 2022a). This demonstrates that AD-related network disruptions extend beyond linear intra-band abnormalities, including impaired cross-frequency non-linear coupling. It underscores the pivotal role of cross-frequency interactions in AD pathophysiology and proposes high-order spectral features as promising novel biomarkers.

## 3 Significance and application of qEEG in diagnosis and differential diagnosis of AD

Different from the traditional EEG, which relies on visual interpretation, qEEG provides objective indicators through linear analysis (such as power spectrum and coherence analysis) and non-linear dynamics (such as sample entropy and bispectrum analysis) to evaluate brain function accurately in AD and other cognitive disorders, qEEG can detect changes in neural synchronization, disconnection, and abnormal oscillation. The potential pathological mechanism reflected by these biomarkers can help to diagnose AD not only accurately but also effectively distinguish other types of cognitive impairment.

### 3.1 QEEG metrics and network analysis

QEEG is increasingly being applied in the diagnosis and differentiation of cognitive impairments, including AD, MCI, and other forms of dementia. Certain key qEEG measures, such as spectral ratios, have high diagnostic accuracy. For instance, Schmidt et al. (2013) reported that the α/θ ratio in the C3 and O1 channels could differentiate AD patients with a sensitivity of 76.4% and specificity of 84.6%, achieving an ROC AUC of 0.92.

Additionally, the eLORETA-based analysis identified distinct patterns in δ and α bands between AD-related mild cognitive impairment (ADMCI) and dementia with Lewy bodies-related mild cognitive impairment (DLBMCI). Moreover, ADMCI patients exhibited abnormalities in the posterior α band, while DLBMCI patients had greater δ band abnormalities. Occipital α2 source activity effectively distinguished ADMCI from NC (Babiloni et al., 2018). The α reactivity, which reflects cholinergic integrity, was significantly decreased in dementia with Lewy bodies (DLB) compared with levels in AD (Schumacher et al., 2020). This finding highlights its application value in distinguishing DLB from AD.

Mehram et al. (2019) applied EEG-based weighted network measures to distinguish AD from DLB. They used the weighted phase lag index and graph theory to analyze brain network patterns in AD, DLB, and Parkinson’s disease dementia (PDD). The study found that DLB showed weaker posterior-anterior connectivity in the β band and greater network segregation in the θ band, whereas AD had reduced α band functional connectivity (Mehram et al., 2019).

In another study, Bejia et al. (2023) investigated and compared AD with vasculopathy (AD+V), AD without vasculopathy (AD-V), DLB, and vascular dementia (VaD) through qEEG spectral analysis and functional connectivity. Their study revealed higher α band power in VaD compared to AD+V and AD-V. Moreover, AD-V demonstrated elevated β2 band power and stronger functional connectivity in the β2 band relative to AD+V, DLB, VaD, and healthy controls (Bejia et al., 2023).

Yu et al. (2016) compared the EEG functional connectivity characteristics of AD patients, behavioral variant frontotemporal dementia (bvFTD) patients, and subjective cognitive decline (SCD) using PLI and minimum spanning tree (MST) methods. The MST method constructs a simplified brain network model by retaining the strongest connections and eliminating redundant pathways. It was observed that bvFTD patients had significantly higher δ-band PLI values, and AD patients had higher θ-band PLI values than bvFTD but lower α-band PLI values. Further analysis revealed that bvFTD patients had frontal network abnormalities, whereas AD patients showed functional connectivity disruption in parieto-occipital regions, suggesting different pathophysiological mechanisms (Yu et al., 2016). Another study showed that the functional connectivity-based classification model was superior to neuropsychological tests in differentiating bvFTD, AD, and NC, especially in identifying bvFTD from AD (Dottori et al., 2017).

Overall, the cumulative evidence underscores the considerable potential of qEEG in AD, offering unique advantages across early detection, differential diagnosis, and disease monitoring. At the same time, qEEG improves the accuracy of differential diagnosis by distinguishing AD from other types of dementia and neurological disorders, thus providing a basis for developing precise treatment plans. Longitudinal qEEG profiles further correlate with cognitive decline rates, providing real-time insights for therapeutic optimization.

Although qEEG has shown promising results, several limitations must be addressed to improve its broader application. For instance, the validity of qEEG in subtypes like frontotemporal dementia (FTD) or mixed dementia requires further validation through additional studies. Moreover, the absence of standardized testing protocols and the need for larger, more diverse datasets hinder its clinical adoption. Overcoming these challenges would significantly enhance the reliability and utility of qEEG in diagnosis, facilitating its integration into routine clinical practice.

### 3.2 Traditional machine learning methods

Machine learning (ML) techniques have shown high potential to improve the diagnosis power of qEEG in AD. Bairagi (2018) combined spectral features with wavelet features and used support vector machines (SVM) and K nearest neighbor (KNN) classifiers to diagnose AD. The accuracy of the joint feature model was 94%, which is significantly better than the results of using spectral features (90%) or wavelet features (88%) alone. In terms of computational efficiency, SVM took 36.12 s in training and 1.28 s in the testing tasks, exceeding the performance of KNN (Bairagi, 2018). This suggests that the combination of spectral and wavelet features can improve the accuracy and reliability of early diagnosis of AD, making it an ideal approach for clinical practice.

Youssef et al. (2021) constructed a machine learning model based on brain functional network markers with an accuracy of 87.2% in MCI diagnosis. However, the model’s generalizability remains constrained by cohort size and inter-site heterogeneity, necessitating validation in more extensive, multi-center populations. Siuly et al. (2020) proposed an EEG analysis method that combines features of arrangement entropy and autoregressive model, and their Extreme Learning Machine (ELM) model significantly outperforms SVM and KNN algorithms in distinguishing MCI, with an overall improvement in accuracy, sensitivity, and specificity and a single operation takes only 0.281 s. This dual integration of non-linear dynamics and linear parametric features effectively captures alterations in EEG complexity associated with MCI. Nonetheless, the method necessitates a rigorous evaluation of its robustness in longitudinal EEG recordings, and its diagnostic sensitivity to preclinical stages of MCI. In addition, Mehram et al. (2019) compared the brain network topology differences of AD, DLB, and PDD by WPLI and graph theory methods, and the random forest analysis showed that the AUC of the distinction between DLB and AD was 78%. This study quantified the specificity of functional connectivity using the Weighted Phase Lag Index (WPLI), underscoring the potential of frequency-domain network properties in differentiating neurodegenerative diseases. Integrating multimodal data, such as structural MRI or biomarkers, could enhance classification performance. Additionally, attention should be given to the impact of graph theory metric selection on model interpretability. These studies demonstrate the significant value of machine learning methods based on brain network features for diagnosing mild cognitive impairment (MCI). Ongoing opportunities exist to address challenges such as algorithm interpretability, cross-dataset robustness, and validating associations with pathological mechanisms.

### 3.3 Deep learning models

Deep learning has demonstrated unique advantages in EEG signal analysis through models such as convolutional neural networks (CNN), recurrent neural networks (RNN), long short-term memory networks (LSTM), and graph neural networks (GNN). Among them, CNN excels at processing spectral or spatial features of qEEG, RNN captures the temporal dynamics of the signal, and GNN models brain functional connectivity networks (Jo et al., 2019; Abadal et al., 2025). Its automated feature extraction capability with high classification accuracy is particularly suitable for high-dimensional non-linear qEEG data analysis, providing new ideas for diagnosing AD and MCI.

Based on the spectral-spatial feature extraction advantages of CNN, Ieracitano et al. (2019) developed a customized CNN model to transform multichannel EEG power spectrum data into channel × frequency grayscale images for distinguishing between AD, MCI, and NC. Through training, the model achieved an AUC value of 0.94 in a triple-classification task, where two-by-two comparisons significantly outperformed conventional methods like SVM (Ieracitano et al., 2019). This study confirms the potential of CNNs to extract discriminative features from EEG signals and provides new ideas for staging the course of dementia.

While CNNs focus on spatial-spectral patterns, the temporal dynamics of EEG require complementary approaches. Gkenios et al. addressed this by integrating convolutional layers (to identify abnormal frequency patterns) with an LSTM network (to analyze temporal variations) for AD/MCI/NC classification. Although temp model achieved 99% accuracy in segment-level validation, its performance dropped to 67% on new patients, highlighting generalization challenges. Nevertheless, the high sensitivity (88.9%) for early cognitive impairment and specificity (88.9%) for AD screening suggest clinical utility, pending larger datasets and multimodal integration (e.g., neuroimaging) (Gkenios et al., 2022).

Recent advances in GNNs show promise for EEG-based AD diagnosis. A study using PSD and functional connectivity (FC) matrices as inputs demonstrated that GNNs achieve superior performance (AUC = 0.984, accuracy = 92%) compared to traditional models (e.g., CNNs with best AUC = 0.924). Dynamically constructed FC-driven graph structures effectively identified AD-related brain network abnormalities, with minimal performance variation across FC methods, highlighting GNNs’ advantages for brain disorder classification (Klepl et al., 2022b).

However, conventional GNNs typically rely on pre-defined graph structures (e.g., FC matrices), which may overlook local spectral-spatial features. To address this limitation, an adaptive gated graph convolutional network (AGGCN) was developed to fuse CNN-extracted node features (from PSD) and connectivity graphs derived from signal correlations. This integration of local and global patterns enabled 89.1% accuracy in classifying AD using eyes-closed EEG (Klepl et al., 2023).

Spatio-temporal integration has emerged as a pivotal frontier. The Spatial-temporal Graph Convolutional Network (ST-GCN) combines wavelet coherence-based functional connectivity (spatial) with 1D convolutions (temporal), achieving 92.3% accuracy in AD classification—surpassing purely temporal models (T-CNN: 88.0%)—while revealing weakened posterior connectivity in AD for mechanistic insights (Shan et al., 2022). However, its reliance on static functional connectivity thresholds risks overlooking subtle network disruptions in heterogeneous dementia subtypes.

In contrast, Adebisi et al. developed a dynamic thresholding method that adaptively optimizes PLI-based networks using eigenvector centrality and healthy cohort references. This approach identified frequency-regional biomarkers: elevated δ band occipital PLI in AD (cortical hyperinhibition) and reduced θ band temporal PLI in MCI (early memory network decline). Integrated with GCNs, their model achieved 95.07% δ-band accuracy in classifying AD, MCI, vascular dementia, and NC, demonstrating robust diagnostic potential for complex neurological disorders (Adebisi et al., 2024).

Current research faces several challenges, including limited model generalizability, which is demonstrated by the sharp accuracy degradation of LSTMs when applied to new patient coorts requiring expanded multi-center datasets to enhance adaptation. Overreliance on single-modality EEG data persists without systematically integrating neuroimaging or biochemical biomarkers, thereby restricting diagnostic comprehensiveness. Dynamic network construction methods, such as dynamic thresholding, are highly complex and require high computation power, making them less applicable in clinical practice. Moreover, limited interpretability, especially regarding the cross-frequency interactions and spatial coupling mechanisms, hinders a deeper understanding of the underlying mechanisms. Future research should prioritize the development of efficient, multimodal-adaptive frameworks that rigorously validate network features about established pathological pathways. Additionally, advancing prospective clinical trials is essential to enhance the practical applicability and translational outcomes. For a summary of published studies on the diagnostic performance of qEEG in AD and MCI, refer to Table 1.

**TABLE 1:** Diagnostic performance of qEEG in Alzheimer’s disease and mild cognitive impairment.

Population	qEEG parameters	AI-model/method	Performance metrics	References
AD 50, NC 57	α/θ	ROC	AD vs. NC: AUC = 0.92, Sen = 76.4%, Spe = 84.6%	Schmidt et al., 2013
AD 50, NC 50	Spectral	SVM	AD vs. NC: Acc = 90%, Sen = 92%, Spe = 88%	Bairagi, 2018
	Wavelet		AD vs. NC: Acc = 88%, Sen = 92%, Spe = 84%	
	Spectral and wavelet		AD vs. NC: Acc = 94%, Sen = 96%, Spe = 92%	
MCI 11, NC 16	Permutation entropy and auto-regressive	ELM	MCI vs. NC: AUC = 0.98, Acc = 98.8%, Sen = 98.3%, Spe = 99.7%	Siuly et al., 2020
		SVM	MCI vs. NC: AUC = 0.95, Acc = 97.4%, Sen = 95.1%, Spe = 100%	
		KNN	MCI vs. NC: AUC = 0.96, Acc = 98.2%, Sen = 100%, Spe = 95.7%	
AD 330, NC 246	Spectral features, Hjorth parameters, sample entropy, microstates	LDA, SVM	AD vs. NC: Acc = 85%	Jiao et al., 2023
MCI 189, NC 246			MCI vs. NC: Acc = 80%	Jiao et al., 2023
AD 330, MCI 189, NC 246			AD vs. MCI vs. NC: Acc = 70%	Jiao et al., 2023
ADMCI 30, DLBMCI 23	eLORETA	ROC	ADMCI vs. DLBMCI: AUC = 0.72, Acc = 72.5%, Sen = 78.3%, Spe = 66.75%	Babiloni et al., 2018
ADMCI 30, NC 30			ADMCI vs. NC: AUC = 0.86, Acc = 81.7%, Sen = 90.0%, Spe = 73.3%	Babiloni et al., 2018
DLBMCI 23, NC 30			DLBMCI vs. NC: AUC = 0.89, Acc = 81.3%, Sen = 82.6%, Spe = 80.0%	Babiloni et al., 2018
AD 13, NC 18	Functional connectivity analysis	SVM	AD vs. NC: AUC = 0.54, Acc = 44.9%	Dottori et al., 2017
AD 13, bvFTD 13	Functional connectivity analysis	SVM	AD vs. bvFTD: AUC = 0.73, Acc = 72.9%	Dottori et al., 2017
DLB 25, AD 32	WPLI and network feature-based approaches	Random forest	DLB vs. AD: AUC = 0.78, Acc = 66%, Sen = 47%, Spe = 100%	Mehram et al., 2019
NC 18, DLB/PDD 46			NC vs. DLB/PDD: AUC = 0.82, Acc = 76%, Sen = 59%, Spe = 100%	Mehram et al., 2019
AD 63, NC 63	PSD-image	CNN	AD vs. NC: AUC = 0.97, Acc = 92.95%	Ieracitano et al., 2019
	Spectral features	SVM	AD vs. NC: AUC = 0.95, Acc = 82.69%	
AD 63, MCI 63	PSD-image	CNN	AD vs. MCI: AUC = 0.93, Acc = 84.62%	Ieracitano et al., 2019
	Spectral features	SVM	AD vs. MCI: AUC = 0.83, Acc = 60.47%	
MCI 63, NC 63	PSD-image	CNN	MCI vs. NC: AUC = 0.97, Acc = 91.88%	Ieracitano et al., 2019
	Spectral features	SVM	MCI vs. NC: AUC = 0.95, Acc = 71.37%	
AD 63, MCI 63, NC 63	PSD-image	CNN	AD vs. MCI vs. NC: AUC = 0.94, Acc = 83.33%	Ieracitano et al., 2019
	Spectral features	SVM	AD vs. MCI vs. NC: AUC = 0.88, Acc = 56.84%	
AD 20, NC 20	AE, PSD	GNN	AD vs. NC: AUC = 0.98, Acc = 91.9%, Sen = 97.4%, Spe = 86.7%	Klepl et al., 2022b
AD 20, NC 20	Mutual information	CNN	AD vs. NC: AUC = 0.92, Acc = 84.7%, Sen = 86.2%, Spe = 83.2%	Klepl et al., 2022b
AD 20, NC 20	Mutual information	SVM	AD vs. NC: AUC = 0.81, Acc = 73.9%, Sen = 73.5%, Spe = 74.4%	Klepl et al., 2022b
AD 18, NC 18	Frequency domain features	LSTM	AD vs. NC: Acc = 83.4%	Gkenios et al., 2022
AD 18, MCI 18			AD vs. MCI: Acc = 80.5%	Gkenios et al., 2022
MCI 18, NC 18			MCI vs. NC: Acc = 80.5%	Gkenios et al., 2022
AD 18, MCI 18, NC 18			AD vs. MCI vs. NC: Acc = 99.0% (Segment-level validation), Acc = 67.0% (subject-level validation)	Gkenios et al., 2022
AD 20, NC 20	Functional connectivity analysis, PSD	AGGCN	AD vs. NC: AUC = 0.895, Acc = 89.1%	Klepl et al., 2023
AD 19, NC 20	Wavelet coherence	ST-GCN	AD vs. NC: Acc = 92.3%	Shan et al., 2022
		TCNN	AD vs. NC: Acc = 88.0%	
AD16, MCI 14, VaD 10, NC 10	PLI-based brain networks	GCN-net	AD vs. MCI vs. VaD vs. NC: Acc = 95.07%	Adebisi et al., 2024

AD, Alzheimer’s disease; NC, normal control; MCI, mild cognitive impairment; DLB, dementia with Lewy bodies; bvFTD, behavioral variant frontotemporal dementia; PDD, Parkinson’s disease dementia; VaD, vascular dementia; qEEG, quantitative electroencephalography; ROC, receiver operating characteristic; AUC, area under the curve; Acc, accuracy; Sen, sensitivity; Spe, specificity; SVM, support vector machine; KNN, K-nearest neighbors; ELM, extreme learning machine; LDA, linear discriminant analysis; WPLI, weighted phase lag index; CNN, convolutional neural network; GNN, graph neural network; LSTM, long short-term memory; AGGCN, adaptive graph convolutional network; ST-GCN, spatial-temporal graph convolutional network; TCNN, temporal convolutional neural network; GCN-net, graph convolutional network; AE, amplitude envelope correlation; PSD, power spectral density; EC, eyes closed; EO, eyes open; PLI, phase lag index.

## 4 Multimodal integration of qEEG with complementary biomarkers

Integrating qEEG with complementary biomarkers, such as MRI, molecular markers like amyloid-beta (Aβ) and tau, genetic factors, and clinical scales, offers a comprehensive approach to understanding and diagnosing neurological disorders. By combining the temporal resolution of qEEG with structural, molecular, and genetic insights, this multimodal framework enhances diagnostic precision, uncovers pathophysiological relationships, and paves the way for non-invasive, holistic tools in neurophysiological and neurodegenerative research. Refer to Table 2 for a summary of the studies exploring the multimodal integration of qEEG and biomarkers in differentiating Alzheimer’s disease, mild cognitive impairment, and other dementias.

**TABLE 2:** Multimodal integration of qEEG and biomarkers for differentiating AD, MCI, and other dementias.

Population	Analysis/parameters	AI-model/method	Performance metrics	References
AD13, NC 18	Functional connectivity analysis	SVM	AD vs. NC: AUC = 0.54, Acc = 44.9%	Dottori et al., 2017
	Functional connectivity analysis+neuropsychological tests		AD vs. NC: AUC = 0.77, Acc = 88.0%	
bvFTD 13, NC 25	Functional connectivity analysis	SVM	bvFTD vs. NC: AUC = 0.73, Acc = 72.7%	Dottori et al., 2017
	Functional connectivity analysis+neuropsychological tests		bvFTD vs. NC: AUC = 0.78, Acc = 87.4%	
AD 13, bvFTD 13	Functional connectivity analysis	SVM	AD vs. bvFTD: AUC = 0.73, Acc = 72.9%	Dottori et al., 2017
	Functional connectivity analysis+neuropsychological tests		AD vs. bvFTD: AUC = 0.70, Acc = 72.9%	
AD 111	Spectral features, Functional connectivity analysis+MRI	SVM	MMSE ≥ 24 vs. MMSE < 24: Acc = 84.7%, Sen = 92.1%, Spe = 75.0%	Waser et al., 2019
	MRI		MMSE ≥ 24 vs. MMSE < 24: Acc = 66.7%, Sen = 77.8%, Spe = 52.1%	
	Spectral features, functional connectivity analysis		MMSE ≥ 24 vs. MMSE < 24: Acc = 79.3%, Sen = 87.3%, Spe = 68.8%	
AD 60, aMCI 60	MRI	Penalized logistic regression	AD vs. aMCI: AUC = 0.99	Farina et al., 2020
	wPLI		AD vs. aMCI: AUC = 0.62	
	MMSE+global theta		AD vs. aMCI: AUC = 0.86	
AD 60, NC 60	MRI		AD vs. NC: AUC = 0.99	Farina et al., 2020
	wPLI		AD vs. NC: AUC = 0.76	
	MMSE+global theta		AD vs. NC: AUC = 0.98	
aMCI 60, NC 60	MRI		aMCI vs. NC: AUC = 0.72	Farina et al., 2020
	wPLI		aMCI vs. NC: AUC = 0.61	
	MMSE+global theta		aMCI vs. NC: AUC = 0.80	
MCI pos 51, MCI neg 35	fMRI, eLORETA	Binomial logistic regression models	MCI pos vs. MCI neg: Acc = 76.8%, Sen = 71.4%, Spe = 70.6%	Cecchetti et al., 2021
AD28, NC 28	DTABR	ROC	AD vs. NC: AUC = 0.782	Niu et al., 2023
	APOA-I,		AD vs. NC: AUC = 0.713	
	APOE4		AD vs. NC: AUC = 0.679	
	DTABR, APOA-I, APOE4		AD vs. NC: AUC = 0.889	
aMCI 29, NC 28	DTABR	ROC	aMCI vs. NC: AUC = 0.833	Niu et al., 2023
	APOA-I		aMCI vs. NC: AUC = 0.669	
	APOE4		aMCI vs. NC: AUC = 0.584	
	DTABR, APOA-I, APOE4		aMCI vs. NC: AUC = 0.855	
AD 30, DLB 21	Spectral features, functional connectivity analysis	SVM	AD vs. DLB: Acc = 77%	Colloby et al., 2016
	MTA		AD vs. DLB: Acc = 67%	
	Spectral features, functional connectivity analysis+MTA		AD vs. DLB: Acc = 90%	

MMSE, mini-mental state examination, a cognitive assessment tool; aMCI, amnestic mild cognitive impairment; DTABR, (δ+θ)/(α+β) relative power ratio; APOA-I, apolipoprotein A-I; APOE4, apolipoprotein E4; MRI, magnetic resonance imaging; MTA, medial temporal atrophy.

### 4.1 Integration of quantitative EEG and magnetic resonance imaging

Abnormal EEG rhythm has been linked to cortical gray matter atrophy and cognitive function (Babiloni et al., 2013a). Studies using LORETA source models reported a significant correlation between decreased hippocampal and occipital gray matter volume and density levels and decreased cortical α power in patients with MCI and AD (Babiloni et al., 2009, 2014). Notably, MCI patients approaching AD conversion showed elevated α3/α2 power ratios, with higher ratios associated with more significant cortical loss in the inferior parietal lobule (exceptionally superior limbic gyrus and bilateral precuneus) (Moretti, 2018). These findings demonstrate a strong correlation between EEG abnormalities and MRI-derived structural changes, such as gray matter atrophy, suggesting that EEG and neuroimaging data can be integrated to provide a more comprehensive understanding of neurodegenerative processes in MCI and AD.

To evaluate the diagnostic utility of MRI-EEG markers, Waser et al. (2019) integrated volumetric data (frontal, parietal, temporal, occipital, entorhinal cortex, hippocampus, and amygdala) with EEG spectral power/coherence measures through machine learning. In AD patients, reduced parietal lobe volume and cortical thickness correlated with elevated delta power and lower β power. This multimodal approach achieved 84.7% accuracy in distinguishing MMSE ≥ 24 from MMSE < 24 patients (Waser et al., 2019). This result highlights the advantages of a multimodal approach, whereby combined structural (e.g., cortical thickness) and functional (e.g., slow-wave activity enhancement) analyses can be more sensitive to capturing early pathological features of AD and compensate for the shortcomings of a single modality.

Memory dysfunction subtypes in MCI show distinct profiles: the encoding dysfunction (EF) group displays increased frontal θ power, decreased β2 power, and enhanced θ connectivity, alongside reduced left thalamic and bilateral hippocampal gray matter volumes. These structural-functional changes align with AD prodromal characteristics compared to retrieval dysfunction (RF) groups (Han et al., 2021). However, the clinical prognostic significance of differentiating EF and RF subtypes, particularly regarding AD conversion rates, is unclear and requires validation through longitudinal studies.

The study by Schumacher et al. (2020) further reveals the potential for differential diagnosis of AD from other disorders based on EEG source activity analysis. Compared to the normal control group, DLB and AD patients exhibited higher δ source activity (DLB > AD) and lower α source activity (AD > DLB), with DLB patients experiencing rapid eye movement (REM) sleep behavior disorder showing reduced central α source activity. As cognitive impairment progresses, increased θ source activity in the parietal and central regions was noted, alongside enhanced α source activity in the central, parietal, and occipital regions. Visual hallucinations were associated with elevated parietal δ source activity, suggesting these features are key markers for distinguishing AD and DLB (Schumacher et al., 2020). Furthermore, researchers developed an EEG-MRI model combining EEG spectral and coherence features with medial temporal lobe atrophy (MTA) scores, achieving a classification accuracy of 90%, outperforming single-modality models (Colloby et al., 2016). This indicates that the EEG-MRI combination enhances diagnostic performance and could potentially replace dopaminergic imaging.

Nevertheless, the generalizability of this model requires cautious evaluation. The high heterogeneity of DLB (e.g., AD co-pathology, REM sleep behavior disorder, or autonomic failure) may compromise the stability of EEG-MRI fusion features. Furthermore, unresolved discrepancies in MRI scanner parameters and EEG preprocessing methods across multicenter studies pose additional challenges. Future research needs to validate the predictive efficacy of these biomarkers for disease progression (e.g., from MCI to DLB/AD) in longitudinal cohorts and elucidate the mechanistic contribution of key features and their causal relationship with pathological mechanisms, thereby advancing multimodal diagnostic tools toward personalized therapeutic strategies.

### 4.2 Unveiling the relationship between qEEG, Aβ, and tau: toward a non-invasive diagnostic tool

Currently, it is particularly important to gain a deeper understanding of the correlation between qEEG and Aβ and tau proteins and their underlying mechanisms. QEEG reflects changes in brain electrical activity patterns, which may be closely related to the accumulation of Aβ and tau. By exploring these relationships, we can reveal the pathological mechanisms of neurodegenerative diseases and provide new perspectives for early diagnosis.

In AD patients, CSF analysis reveals significantly decreased Aβ42 levels and increased total tau (t-tau) and phosphorylated tau (p-tau) levels (Blennow et al., 2010). Smailovic et al. (2018) reported a negative correlation between CSF Aβ42 levels and slow-frequency power (θ and δ), as well as between p-tau and t-tau levels and fast-frequency power (α and β). Reduced CSF Aβ42 and elevated p-tau and t-tau were also associated with decreased α and β GFS levels. Mechanistically, amyloid pathology may cause slow-frequency interference through the cholinergic system, while tau pathology, akin to neuronal loss and degeneration, affects fast frequencies in the cortex (Smailovic and Jelic, 2019).

Rochart et al. (2020) investigated gamma activity in cognitively normal patients with abnormal CSF amyloid and tau ratios. They found impaired γ-band power in individuals with positive AD biomarkers (Rochart et al., 2020). Similarly, (Kim et al., 2021) categorized subjective cognitive decline (SCD) and MCI patients into amyloid-positive and amyloid-negative groups using PET-CT. The amyloid-positive group exhibited higher δ absolute power and δ relative power, as well as higher β1 relative power. Conversely, γ absolute power, α relative power, and γ relative power were lower in the amyloid-positive group (Kim et al., 2021). Cecchetti et al. (2021) grouped MCI patients based on CSF tau and Aβ42 ratios (MCIpos ≥ 0.13, MCIneg < 0.13). AD and MCIpos patients showed higher θ density in the central, parietal, and occipital lobes compared to MCIneg and healthy individuals (Cecchetti et al., 2021).

QEEG parameters, particularly δ and θ GFS, provide critical insights into AD pathology. Studies reveal that reduced δ/θ GFS in patients with single-domain amnestic mild cognitive impairment (sd-aMCI) mirrors neurophysiological changes seen in AD, including synaptic damage, amyloid pathology, and neurodegeneration. These qEEG patterns further highlight impaired slow-band functional connectivity, which is strongly associated with early AD manifestations (Smailovic et al., 2022). However, the cross-sectional nature of existing studies has limited the ability to fully investigate the dynamic relationship between qEEG features and the progression of Aβ/Tau pathology. To address this, future research should prioritize longitudinal cohort studies to systematically track changes in qEEG parameters alongside biomarker evolution and cognitive decline. Integrating multimodal data, such as structural MRI, plasma biomarkers, and tau-PET imaging, will further enhance diagnostic accuracy and deepen our understanding of the underlying pathological mechanisms, ultimately advancing precision medicine approaches for neurodegenerative diseases.

### 4.3 Integration of qEEG and other examinations

Apolipoprotein A-I (APOA-I) modulates AD occurrence and progression by binding to Aβ (Zuin et al., 2021), while the apolipoprotein E (APOE4) allele serves as a potential AD biomarker (Corder et al., 1993). Combining DTABR with APOA-I and APOE4 optimizes aMCI diagnostic accuracy (Niu et al., 2023). While APOE4 does not directly drive AD-related EEG slowing, it is linked to selective reductions in EEG coherence (Jelic et al., 1997). However, a large-scale study observed more severe EEG slowing in AD patients without the APOE4 allele, particularly in parieto-occipital regions (de Waal et al., 2013). Additionally, APOE4 carriers with amyloid pathology exhibit higher β band GFP and lower θ and β band GFS than non-carriers (Smailovic et al., 2021). The increased β band GFP in APOE4 carriers may reflect compensatory mechanisms to counteract global EEG slowing, while reduced θ and β band GFS indicate brain functional deficits linked to the APOE4 genotype.

Farina et al. (2020) demonstrated that magnetic resonance imaging (MRI) achieved superior accuracy in distinguishing AD patients from NC (AUC = 0.99) and AD from MCI (AUC = 0.99) but showed lower performance in differentiating MCI patients from NC (AUC = 0.73). Standalone EEG showed limited discrimination between MCI and NC groups (AUC = 0.61), yet integration with the Mini-Mental State Examination (MMSE) improved AUC to 0.80, confirming the augmentative value of behavioral metrics in EEG-based diagnosis (Farina et al., 2020). Dottori et al. (2017) revealed context-dependent benefits of neuropsychological variables (NPVs): NPVs enhanced EEG connectivity models for distinguishing behavioral variant frontotemporal dementia (bvFTD) patients from healthy controls (87.4% vs. 72.7% accuracy) but provided no advantage for differentiating bvFTD from AD. Connectivity variables alone achieved 88.3% accuracy in distinguishing AD patients from healthy controls unaffected by NPV integration (Dottori et al., 2017). This demonstrates that connectivity biomarkers have higher disease-specific discriminative capacity than behaviorally-enhanced models.

Integrating qEEG with other data types is undoubtedly set to become a key trend in future research. However, the heterogeneity of multimodal data, including the static nature of genetic or molecular biomarkers (e.g., APOE4 genotype or Aβ/Tau protein concentrations), the spatial specificity of MRI, the subjectivity of scale assessments, and the high temporal resolution dynamic characteristics of EEG, significantly increases the complexity of algorithmic integration, limiting the development of individualized predictive models. Future efforts must focus on developing cross-scale fusion algorithms to integrate multimodal data, analyze time-space-functional relationships, and construct precise, interpretable, individualized predictive models.

## 5 Application value of qEEG in the prediction of AD progression

Multiple studies underscore the growing utility of qEEG in predicting AD progression, particularly in individuals with MCI (Engedal et al., 2020; Hamilton et al., 2021; Kim et al., 2021). Hamilton et al. (2021) demonstrated that an elevated θ/α ratio on qEEG was associated with a higher annual risk of dementia in both MCI patients and normal controls over an average follow-up period of 1.5 years, highlighting qEEG’s potential as a dynamic biomarker for early risk stratification. To enhance prediction accuracy, Hatz et al. (2015) tracked 35 aMCI and mild dementia patients over 30 months, identifying significant θ-band PLI differences between left centro-lateral and parieto-occipital brain regions via EEG microstate analysis. Combined with verbal memory scores, this approach achieved 100% specificity and 77% sensitivity in distinguishing AD progressors from stable aMCI (Hatz et al., 2015). This demonstrates that cognitive testing can help to clarify the predictive power of qEEG, although it has a relatively lower sensitivity. This necessitates the use of several biomarkers to reduce false negatives.

The integration of machine learning (ML) has further refined predictive models. One study evaluated qEEG using the statistical pattern recognition (SPR) method to predict dementia conversion in SCD and MCI patients. Over 5 years, 70 of 213 participants developed dementia. The EEG-derived dementia index (DI) showed moderate predictive power (AUC 0.78, 71% sensitivity, 69% accuracy), improving with cognitive tests. qEEG can aid in identifying high-risk patients but should supplement standard diagnostics (Engedal et al., 2020). Multimodal approaches have shown even more tremendous promise in overcoming the limitations of single-modality predictors. Jiao et al. (2023) developed a Random Forest regression model integrating EEG biomarkers, cerebrospinal fluid (CSF) biomarkers, APOE genotype, and demographic data, which outperformed traditional CSF+APOE models in predicting disease onset and progression, particularly for cognitive assessments like MMSE and MoCA. Similarly, while MRI features alone surpassed EEG in predicting AD severity, a study by Jesus et al. (2021) highlighted enhanced accuracy when combining MRI and EEG.

Importantly, recent advances have addressed the need for validation against gold-standard biomarkers. Kim et al. (2021) developed an EEG-ML algorithm for detecting Aβ pathology in SCD and MCI patients, validating its performance against Aβ-PET with 88.6% accuracy in SCD and 84.6% accuracy in MCI amyloid classification, thereby proving the standalone utility of quantitative EEG in identifying Aβ accumulation. Although integrating qEEG with neuropsychological testing, multimodal biomarkers, and machine learning has shown great potential to improve personalized AD prediction, critical challenges remain to be solved, as shown in Table 3. Most previous studies focused on short-term outcomes; thus, the long-term prognostic value is poorly understood. Moreover, the high heterogeneity of EEG feature selection and machine learning methodologies limits cross-study comparisons. Future longitudinal multimodal cohorts, standardized analytical frameworks, and translational studies are needed to resolve and address the current limitations.

**TABLE 3:** QEEG-based unimodal and multimodal approaches for predicting Alzheimer’s disease progression.

Population	Prediction target	Analysis/parameters	AI-model/method	Performance metrics	References
SCD 180	Aβ+ vs. Aβ−	EEG	SVM	Acc = 88.6%, Sen = 85.7%, Spe = 89.3%	Kim et al., 2021
MCI 63	Aβ+ vs. Aβ−		SVM	Acc = 84.6%, Sen = 83.3%, Spe = 85.7%	Kim et al., 2021
SCD 47, MCI 99, NC 67	conversion to AD	EEG	SPR	AUC = 0.78, Acc = 69%, Sen = 71%, Spe = 69%	Engedal et al., 2020
AD 330, MCI 189, NC 246	MMSE	EEG	Random forest regression	MAE = 2.67, R^2^ = 0.82	Jiao et al., 2023
		CSF+APOE		MAE = 2.09, R^2^ = 0.87	
		EEG+CSF+APOE		MAE = 1.69, R^2^ = 0.93	
AD 330, MCI 189, NC 246	MoCA	EEG	Random forest regression	MAE = 2.32, R^2^ = 0.85	Jiao et al., 2023
		CSF+APOE		MAE = 1.44, R^2^ = 0.94	
		EEG+CSF+APOE		MAE = 0.88, R^2^ = 0.98	
AD 330, MCI 189, NC 246	Age of onset	EEG	Random forest regression	MAE = 2.95 year, R^2^ = 0.87	
		CSF+APOE		MAE = 4.36 year, R^2^ = 0.65	
		EEG+CSF+APOE		MAE = 1.53 year, R^2^ = 0.95	
AD 330, MCI 189, NC 246	Course of disease	EEG	Random forest regression	MAE = 0.87	
		CSF+APOE		MAE = 1.12	
		EEG+CSF+APOE		MAE = 0.89	
minimal-mild AD 89	MMSE	EEG	Random forest regression	RMSE = 1.798 ± 0.176	Jesus et al., 2021
		MRI		RMSE = 1.715 ± 0.186	
		EEG+MRI		RMSE = 1.682 ± 0.177	
aMCI 35	MCI-stable (*n* = 9), AD (*n* = 26)	EEG+verbal learning memory score	binary logistic regression	AUC = 0.90, Sen = 77%, Spe = 100%, Positive predictive value = 100%, Negative predictive value = 60%	Hatz et al., 2015

SCD, subjective cognitive decline; Aβ+, amyloid beta positive; Aβ−, amyloid beta negative; SPR, statistical pattern recognition; MoCA, Montreal cognitive assessment; MAE, mean absolute error; R^2^, coefficient of determination; RMSE, root mean squared error.

## 6 Application of qEEG in monitoring disease treatment in patients with MCI and AD

QEEG biomarkers have demonstrated promise in managing neurodegenerative diseases, particularly AD, by providing real-time, non-invasive insights into synaptic dysfunction and neurochemical imbalances associated with disease progression.

Decreased acetylcholine synthesis or increased acetylcholine breakdown may contribute to the development of AD (Bartus et al., 1982). A potential strategy to enhance cholinergic neurotransmission is to increase the availability of acetylcholine by inhibiting acetylcholinesterase (AChEI). Several acetylcholinesterase inhibitors, such as Donepezil and Galantamine, have been approved for treating AD (Blennow et al., 2006). Recent studies have shown that AChEI decreases the β, θ, and δ bands in patients with AD. At the same time, conflicting results have been reported in the α band, where Donepezil and Galantamine decrease the α band power, while Rivastigmine increases the α band power (Arjmandi-Rad et al., 2023). This limitation may stem from the fact that most studies investigating the effects of AChEI are based on small samples and lack uniformity in the use of qEEG-related parameters and methods. Therefore, in the future, it is necessary to conduct further studies with a larger number of participants and compare the application of different qEEG parameters to assess the effects of AChEI more comprehensively, thus improving the reliability and validity of the studies.

Other pharmacological interventions have shown beneficial effects on qEEG profiles in AD patients. In a 12-week randomized trial by Scheltens et al. (2018), cognitively impaired patients (*n* = 120) received either PQ912, a glutamine cyclase inhibitor that reduces neurotoxic pyroglutamate-Aβ oligomers, or a placebo beverage. PQ912-treated patients exhibited improved memory function and decreased θ band power, highlighting qEEG’s role in monitoring Aβ-related network modulation (Scheltens et al., 2018). Despite these promising results, the study faced limitations, including unstratified baseline qEEG characteristics and the absence of long-term follow-up beyond 6 months.

Fosgonimeton, a neurotrophic agent that reverses synaptic discontinuity and neuronal loss, has demonstrated the potential to alleviate dementia symptoms and slow disease progression. Clinical studies have shown that Fosgonimeton induces dose-dependent increases in γ power (20–90 mg dose range), correlating with improvements in cognitive function. These findings highlight the effectiveness of qEEG signals in guiding dose optimization in human clinical trials (Hua et al., 2022). However, there are methodological gaps, and the changes in γ power were not validated against synaptic density biomarkers, which reduces the reliability of the results.

In animal studies, the Trk receptor modulator ACD856 enhanced cognitive function and induced dose-dependent qEEG changes, including an increased θ/β ratio, elevated θ power, and reduced α and β power. Importantly, ACD856 has been demonstrated to effectively cross the blood-brain barrier, achieving relevant exposure levels in the central nervous system (CNS) (Önnestam et al., 2023). These findings collectively suggest that its CNS penetration and modulation of qEEG parameters may underlie its cognitive-enhancing effects, making it a promising candidate for developing therapeutic interventions for neuropsychiatric and cognitive disorders.

Transcranial direct current stimulation (tDCS), a non-invasive, non-pharmacological intervention, has been found to improve cognitive function. When left prefrontal cortex (PFC) anodic stimulation (atDCS) and cathodic stimulation (ctDCS) were administered to 7–8 month-old APP/PS1 transgenic AD mice for five consecutive days (20 min per day), memory function was significantly improved and correlated with the decrease of δ-wave and the increase of γ-wave in the EEG activity of the PFC (Duan et al., 2022). This result indicates that tDCS can alleviate brain activity retardation and enhance cognitive performance in AD model animals, suggesting that it may be a robust intervention for restoring neural network activity and AD treatment (Duan et al., 2022). These findings demonstrate that tDCS can restore aberrant neural oscillations in AD mouse models, improve cognitive performance, and underscore its therapeutic potential for normalizing network-level neural dynamics in AD.

Several machine learning algorithms have been used to predict the neurophysiological response of AD patients to tDCS and cognitive interventions. Among them, the machine learning model constructed by Andrade et al. (2023) screened five brain regions, including FC1, F8, CP5, Oz, and F7, for electrical activity as the best predictors of response to tDCS treatment, suggesting that these regions may become biomarkers for treatment prediction. Marceglia et al. (2016) further explored the mechanism of tDCS action by recording EEG before eye closure and 30 min after receiving anodic/cathodic tDCS stimulation and assessing working memory changes in conjunction with a word recognition task (WRT). Spectral power and EEG coherence across frequency bands were analyzed. Baseline results showed reduced high-frequency power correlated with lower MMSE scores. After atDCS, high-frequency power and coherence increased in the temporoparietal and temporoparietal-occipital regions, respectively, aligning with improved WRT performance. In contrast, ctDCS reduced theta power across the scalp without clinical correlation. These findings suggest that atDCS reverses abnormal brain activity in AD, improving working memory through cortical regulation (Marceglia et al., 2016). Although tDCS can improve cognitive performance in AD, its clinical application is limited by the lack of standardized protocols, long-term data, and constraints of translating findings from animal models to humans.

In summary, qEEG performs well in monitoring treatments for MCI and AD, but further research is needed to address the various gaps shown in Table 4. In the future, large-scale multicenter clinical trials should test the reliability of qEEG in different populations. In addition, the intervention plan for qEEG should be adjusted using real-time treatment monitoring, thereby improving individualized efficacy. Researchers should develop standard data acquisition methods and biomarker interpretation protocols. Future research should focus on integrating qEEG with advanced neuroimaging modalities such as MRI and PET to enable a more comprehensive analysis of disease mechanisms and the identification of potential therapeutic targets. Moreover, further exploration of emerging computational approaches, particularly machine learning, could facilitate the discovery of novel biomarkers within qEEG data, revealing neural activity patterns that may be difficult to capture using traditional methods. Moreover, applying machine learning for biomarker identification could enhance the understanding of treatment response dynamics and disease progression, ultimately contributing to developing more effective and personalized therapeutic strategies for MCI and AD.

**TABLE 4:** Summary table of studies on the application of qEEG in monitoring disease treatment across populations and models.

Study population	Intervention measures	qEEG parameters	EEG assessment time points	Results	Main conclusions	References
AD (*n* = 50)	Galantamine	α, β and θ power	5 h	Decrease in frontal α, β and θ power	The acute pharmacodynamic effects (reduction in α, β and θ power) following a single dose of 16 mg galantamine are associated with long-term therapeutic outcomes.	Baakman et al., 2022
AD (*n* = 20)	Rivastigmine	High α band and low α band power	Baseline and 18 months post-intervention	Percentage of patients with increased α power at P3 electrode: RV-TDP group 80%, RV-CP group 30%	Compared to RV-CP treatment, RV-TDP led to increased posterior α power in more patients and significantly improved cognitive function at 18 months.	Moretti et al., 2014
AD (*n* = 20)	Rivastigmine	θ power	1 week	Reduction in θ power	Reduction in θ power during treatment is a strong predictor of therapeutic response.	Adler et al., 2004
AD (*n* = 18)	Donepezil	Spectral analysis	2 months and 4 months	Temporal δ power decreased, other frequencies increased	Donepezil positively impacted the brain activity of AD patients by reducing δ activity and increasingα and β activity. The post-treatment increase in θ activity may reflect a therapeutic shift fromδ to θ activity.	Balkan et al., 2003
AD (*n* = 12)	Donepezil	Spectral analysis	1 month	A reduction in α band activity across the bilateral frontal-central-temporal cortex and δ band activity over the bilateral frontal region.	Donepezil influences EEG changes related to cognition in AD patients.	Reeves et al., 2002
MCI/AD (*n* = 120)	Glutaminyl cyclase inhibitor PQ912	α and θ power	12 weeks	PQ912 group θ power decreased, placebo group θ power increased.	PQ912 significantly reduced θ band relative power in EEG, indicating its modulatory effect on brain electrical activity.	Scheltens et al., 2018
Healthy young (*n* = 48), elderly volunteers (*n* = 29).	Fosgonimeton	Spectral analysis	Single-dose: Pre and 1 h post-administration (healthy young). Multiple-dose: Pre, 1 h, 3 h on Days 1, 4, 8 (elderly).	Acute and sustained increase in γ power	The effectiveness of qEEG signals in guiding dose optimization in human clinical trials.	Hua et al., 2022
Healthy subjects (*n* = 24) in three groups: 10, 30, 90 mg/day.	ACD856	Spectral analysis	Baseline, 1.5, 6, and 24 h after the first dose, and 1.5 h after the last dose on day 7.	Increased θ power, decreased α and β power, and increased θ/β ratio.	The qEEG parameters demonstrated dose-dependent changes with variations in CD856 dosage.	Önnestam et al., 2023
Mice (7–8 months)	tDCS	Spectral analysis	Spontaneous EEG recorded pre-tDCS and twice post-tDCS; task-related EEG during Y-maze.	atDCS increased PFC α during spontaneous states; ctDCS enhanced α and β during tasks. Improved memory retrieval correlated with PFC δ reduction and γ increase.	tDCS reverses sluggish brain activity in AD mice, leading to further cognitive improvement.	Duan et al., 2022
AD (*n* = 7)	tDCS	Spectral analysis, coherence analysis	Baseline and 30 min post-treatment.	Baseline: AD patients showed reduced high-frequency power, correlating with lower MMSE. atDCS increased temporoparietal high-frequency power and coherence, linked to WRT improvement. ctDCS reduced δ/θ power, but unrelated to WRT outcomes.	atDCS partially reversed abnormal EEG patterns, potentially through the modulation of cortical activity	Marceglia et al., 2016

RV-TDP, rivastigmine transdermal patch; RV-CP, rivastigmine capsule or oral patch; P3, parietal electrode; PFC, prefrontal cortex; tDCS, transcranial direct current stimulation; atDCS, anodal transcranial direct current stimulation; ctDCS, cathodal transcranial direct current stimulation; WRT, word recall test.

## 7 Conclusion

Compared with existing review papers in this field (Cassani et al., 2018; Sanchez-Reyes et al., 2021; Chetty et al., 2024), this manuscript explores qEEG-related parameters in AD and MCI diagnosis, treatment monitoring, and multimodal data fusion, while highlighting advancements in machine learning and deep learning for EEG analysis. These contributions provide a comprehensive reference for AD research, addressing gaps in the field. However, variability in EEG data collection—such as the number of electrodes, sampling time, and recording conditions—and the reliance on manual artifact removal methods, like visual examination, introduce subjectivity and hinder comparability across studies. Automated artifact removal techniques, such as independent component analysis (ICA) or wavelet-based methods, could address these limitations by providing more standardized and reproducible results. To establish qEEG as an indicative biomarker—a marker that provides direct diagnostic evidence rather than supplementary information—further comprehensive studies and standardizing EEG data collection and experimental protocols are essential.

Incorporating ML into qEEG studies is critical for identifying specific data features. Deep learning models such as CNN and GNN in machine learning can efficiently extract spatiotemporal and topological features of EEG signals, while SVM is good at high-dimensional data classification, and these techniques have been successful in epilepsy detection and Parkinson’s disease diagnosis, which are worthwhile for qEEG research. Based on this, high-precision classification models can be developed to distinguish NC, MCI, AD, and other dementia subtypes. In addition, qEEG can directly reflect the impairment of early synaptic activity in AD, which should be incorporated into the design of new drug clinical trials to enhance the efficiency of drug development.

In recent studies, multimodal fusion is increasingly applied, demonstrating that combining qEEG with other biomarkers, such as MRI, PET-CT, and CSF analysis, may yield better results. In addition, it has been shown that multimodal approaches provide better diagnostic accuracy, specificity, and overall performance compared to unimodal approaches. However, current studies often suffer from tiny sample sizes and short follow-up durations, which limit the generalizability and robustness of findings. Future trials should aim for sample sizes exceeding 300 participants and more than one-year follow-up durations to address these limitations. Such large-scale, longitudinal studies are essential to evaluate the stability and predictive power of qEEG biomarkers in a multimodal framework and to ensure reliable and clinically meaningful results.

Finally, numerous prospective studies have demonstrated the potential of qEEG in improving disease diagnosis and monitoring prognosis. However, the consistency of qEEG in detecting PET-CT and CSF-specific neuropathologic changes should be tested in preclinical and clinical AD patients to obtain reliable and robust qEEG measurements that can be used to assess dementia at the individual level. Specific challenges in applying ML to qEEG data include the need for large, well-annotated datasets to train models effectively and the importance of robust cross-validation techniques to ensure generalizability. With continued advancements in technology, standardization of protocols, and integration of machine learning, qEEG has the potential to revolutionize the diagnosis and management of dementia, paving the way for earlier interventions and improved patient outcomes. Future research should address these challenges to establish qEEG as a reliable and clinically actionable biomarker in the fight against AD and related dementias.
